# Community-based implementation and effectiveness in a randomized trial of a risk reduction intervention for HIV-serodiscordant couples: study protocol

**DOI:** 10.1186/1748-5908-9-79

**Published:** 2014-06-20

**Authors:** Alison B Hamilton, Brian S Mittman, John K Williams, Honghu H Liu, Alicia M Eccles, Craig S Hutchinson, Gail E Wyatt

**Affiliations:** 1 UCLA Department of Psychiatry and Biobehavioral Sciences, 760 Westwood Plaza, 38-240 NPI, Box 175919, 90024-1759 Los Angeles, CA, USA; 2 VA Greater Los Angeles Healthcare System, 16111 Plummer Street, 91343 Sepulveda, CA, USA; 3 UCLA Department of Medicine, 760 Westwood Plaza, 90024-1759 Los Angeles, CA, USA

**Keywords:** Implementation science, Hybrid design, HIV prevention, Serodiscordance, Couples, African Americans, Behavioral intervention, Sustainability

## Abstract

**Background:**

The HIV/AIDS epidemic continues to disproportionately affect African American communities in the US, particularly those located in urban areas. Despite the fact that HIV is often transmitted from one sexual partner to another, most HIV prevention interventions have focused only on individuals, rather than couples. This five-year study investigates community-based implementation, effectiveness, and sustainability of ‘Eban II,’ an evidence-based risk reduction intervention for African-American heterosexual, serodiscordant couples.

**Methods/design:**

This hybrid implementation/effectiveness implementation study is guided by organizational change theory as conceptualized in the Texas Christian University Program Change Model (PCM), a model of phased organizational change from exposure to adoption, implementation, and sustainability. The primary implementation aims are to assist 10 community-based organizations (CBOs) to implement and sustain Eban II; specifically, to partner with CBOs to expose providers to the intervention; facilitate its adoption, implementation and sustainment; and to evaluate processes and determinants of implementation, effectiveness, fidelity, and sustainment. The primary effectiveness aim is to evaluate the effect of Eban II on participant (n = 200 couples) outcomes, specifically incidents of protected sex and proportion of condom use. We will also determine the cost-effectiveness of implementation, as measured by implementation costs and potential cost savings. A mixed methods evaluation will examine implementation at the agency level; staff members from the CBOs will complete baseline measures of organizational context and climate, while key stakeholders will be interviewed periodically throughout implementation. Effectiveness of Eban II will be assessed using a randomized delayed enrollment (waitlist) control design to evaluate the impact of treatment on outcomes at posttest and three-month follow-up. Multi-level hierarchical modeling with a multi-level nested structure will be used to evaluate the effects of agency- and couples-level characteristics on couples-level outcomes (*e.g.*, condom use).

**Discussion:**

This study will produce important information regarding the value of the Eban II program and a theory-guided implementation process and tools designed for use in implementing Eban II and other evidence-based programs in demographically diverse, resource-constrained treatment settings.

**Trial registration:**

NCT00644163

## Background

The HIV/AIDS epidemic continues to disproportionately affect African American communities in the US, particularly those located in urban areas [1,2]. Of all racial/ethnic groups in the United States, African Americans have experienced the greatest burden due to HIV/AIDS, accounting for greatest proportion of HIV infections at all stages of the disease. HIV/AIDS remains a health disparity, with Africans Americans representing 44% of all new HIV infections among adults and adolescents (aged 13 years or older) in 2010, while only representing 12% to 14% of the population. In addition, some of the highest rates of sexually transmitted infections (STIs) are found among African Americans, further heightening risk for HIV transmission [3-5].

There is growing evidence that HIV prevention among African American populations is best accomplished within the context of risk for transmission among couples because relationship dynamics and cultural beliefs sometimes contradict HIV prevention messages [6,7]. Research has documented low rates of condom use among some African Americans with steady partners [8], among African American HIV-positive women [9-11], and among the HIV-negative partners of HIV-positive African American women [11,12]. Among heterosexual relationships, African American women’s decisions to negotiate condom use are often influenced by their desire for a partner and a long-term relationship [13]. Risks for HIV infection can be heightened when either partner does not accurately perceive their risks for infection, when concurrent partners exist outside of the primary relationship, or when one partner is unable to negotiate gender and power dynamics in a relationship [14]. Therefore, HIV prevention interventions need to target sexual behavior change in the context of the relationships where risk for transmission occurs.

Recognizing the importance of relationship dynamics, a small number of couples-based interventions have been developed and found to be efficacious in reducing risky sexual behaviors, increasing condom use, reducing STI/HIV transmission, and sustaining these outcomes [15,16]. However, these interventions have not focused specifically on heterosexual African Americans and their disproportionate HIV risks. The National Institute of Mental Health (NIMH)-funded Eban risk reduction intervention was designed to fill that gap. Guided by both social cognitive and culturally-grounded theories [17-20], the Eban intervention is delivered by trained male–female dyads in individual, couple-specific, and group sessions. A cluster randomized trial tested the efficacy of Eban with 535 couples in four U.S. cities [21]. Participants in the intervention condition reported significantly reduced rates of unprotected sex and increased rates of condom use at post-test, six- and 12-month follow-ups, compared to the control condition [21]. These findings offered a strong foundation for subsequent clinical effectiveness and implementation studies to generate additional evidence regarding Eban intervention effectiveness in routine practice settings, and to design and evaluate implementation strategies intended to facilitate adoption of the Eban intervention by community agencies and other appropriate service delivery organizations.

The sequence of research activities described above is suggested by prevailing frameworks guiding the design of integrated programs of clinical and health promotion research, suggesting a sequence of studies to develop an innovative clinical program, test and refine the program through clinical efficacy and effectiveness studies, and then facilitate its implementation, sustainment and spread. These frameworks note that clinical efficacy and effectiveness studies should be followed by pre-implementation research to assess barriers and facilitators to program adoption, followed by pilot implementation studies and larger trials to evaluate multi-component programs to facilitate routine program adoption in diverse settings [22,23]. Thus, achieving the long-term goal of facilitating large-scale implementation of Eban in agencies that serve HIV-positive and at-risk African Americans requires a solid foundation of effectiveness evidence, insights into barriers and facilitators to adoption and implementation of Eban with high fidelity, and evidence of the effectiveness of specific strategies to facilitate adoption.

Although traditional ‘phased’ or ‘pipeline’ models of clinical research suggest a sequence of separate research activities (clinical efficacy, clinical effectiveness, pre-implementation, implementation, scale-up/spread), researchers are increasingly recognizing the need to combine phases to reduce the time and resources required to develop innovative clinical programs, establish their effectiveness, and facilitate their widespread adoption and benefit [24]. Recognizing the considerable time delays involved in completing each distinct phase in this sequence of research activities, Curran *et al*. [25] described hybrid effectiveness-implementation study designs that combine multiple research activities into a single study to expedite progress across the research pipeline. With its hybrid design, this project will offer guidance to future researchers interested in moving rapidly but thoughtfully from efficacy and effectiveness research into implementation research, particularly in resource-constrained settings where implementation barriers may differ from those seen in efficacy studies. Specific Aims are as follows:

### Primary implementation aims

#### Aim one

To assist 10 community-based organizations (CBOs) in implementing and sustaining an evidence-based intervention for HIV serodiscordant African American couples; specifically, to partner with CBOs to expose providers to the intervention through training, facilitate its adoption and implementation, and sustain practice.

#### Aim two

To evaluate processes and determinants of Eban implementation and Eban clinical effectiveness to strengthen the clinical intervention and its implementation by:

1. Assessing acceptability of the intervention, and barriers and facilitators to its implementation;

2. Examining key determinants of fidelity;

3. Understanding how the project’s implementation strategies and tools affect adoption, fidelity, and effectiveness; and

4. Examining key determinants of sustainability.

### Primary effectiveness aim

#### Aim three

To evaluate the effect of intervention implementation on participant outcomes, specifically incidents of protected sex and proportion of condom use.

### Secondary aim

To determine the cost-effectiveness of implementation of Eban II, as measured by implementation costs and potential cost savings associated with a couples-based intervention.

The study incorporates several innovative features. First, planning, governance, and specific implementation activities will be conducted in full partnership between the research team and community stakeholders, to facilitate sustainability following completion of the grant-funded project. The implementation process will involve the State of California Implementation Network (SCIN), a reciprocal, multidirectional information and technology exchange between the research team and the collaborating CBOs, an approach designed to foster effective agency adoption [26]. Second, implementation of Eban II in multiple geographically, culturally, and functionally disparate agencies requires grounding in organizational change theory because participating agencies are being asked to embrace a new evidence-based, couples-focused intervention approach. Like individual behavior change, organizational change is notoriously difficult [27]. Historically, interventions introduced into clinical settings with little attention to the context have generally met with resistance and limited success. Greater attention is now being paid to strategies for supporting organizational change, particularly by focusing on identifying barriers and facilitators to implementation and using this information to refine implementation strategies [28,29]. For example, organizational ‘readiness for change’ [30], as well as staff expectations, perceptions of their workload, and attitudes toward evidence-based practices may encourage or inhibit adoption of these practices [31], and thus are critical to investigate and address [32]. In this study, it will also be critical to examine the broader context in which the participating agencies are situated, as the pressures that these agencies face may well impact their unique and collective capacity to deliver and sustain evidence-based practices. Third, this implementation study is organized conceptually around the Texas Christian University (TCU) Program Change Model (PCM) [33], which was developed to guide the process of transferring research into practice [34]. The PCM involves four action phases: training, adoption, implementation, and practice improvement. Fourth, the project incorporates a nine-month sustainability period in which time research team involvement in site-level activities will be limited to ‘arms-length’ evaluation, without any intervention or implementation-related activities. As recently noted in a systematic review, very little research has examined the extent, nature, or impact of adaptations to interventions that have been implemented [35]. This project will serve to fill a gap in our knowledge about sustainability as it unfolds prospectively. Finally, this project is unique in its incorporation of a cost-effectiveness analysis during the course of implementation. This analysis stands to contribute substantially to the ways in which costs affect and are impacted by community-based implementation of evidence-based practices.

## Methods/design

### Overview of study design

Using an effectiveness/implementation hybrid type II research design [25], this study will investigate factors associated with successful implementation of Eban in a sample of routine service delivery settings, and real-world clinical effectiveness of the intervention as it is delivered to 180 couples in these settings.

To achieve our two implementation evaluation aims (aims one and two), a mixed methods evaluation will be conducted throughout each phase of the TCU Program Change Model. To achieve our intervention effectiveness aim (aim three), we will randomly assign couples in a 2:1 design to Eban II (intervention) or to a waitlist comparison condition in each of the participating CBOs. This design allows us to compare the relative effectiveness of the intervention with couples that receive the intervention immediately vs. those who are waitlisted.

We operationalize ‘successful implementation’ [36] as a combination of number of couples served (minimum of 18 couples per agency), three completed cycles of the intervention, delivery of the intervention with high fidelity, and high level of satisfaction with the intervention. As noted above, we will also be studying sustainability and cost effectiveness of the intervention for couples. Staff and organizational characteristics will be assessed at all participating CBOs in the first year, and engagement and training will be priorities. Following successful training, implementation of Eban II will commence in two randomly selected CBOs in the first year. The remaining eight CBOs will be phased in randomly with refresher trainings in years two through five. At first glance, it might seem optimal to start all participating agencies at the same ‘baseline,’ but the structure of the funding mechanism makes such a design prohibitive due to the costs associated with implementation in each agency. By limiting training to two to three CBOs per year, we will be able to devote sufficient effort to test whether our implementation approach does in fact contribute to high fidelity implementation. Also, this randomized ‘roll-out’ implementation, or dynamic wait-listed design, has very good statistical properties, including higher power than traditional wait-listed designs [37] and less vulnerability to external, uncontrolled factors [38]. After each of the collaborating agencies completes three eight-week intervention cycles, sustainability will be studied for nine months, during which time only technical assistance will be provided, as discussed further below.

### Participating agencies and staff

HIV infection rates in California rank third in the U.S.; Los Angeles and Alameda Counties have the largest concentrations of African Americans in California and, accordingly, the highest proportion of African American HIV/AIDS cases [39]. A recent report indicates that the City of Oakland (Alameda County) has the highest and most rapidly growing incidence rate of diagnosed and undiagnosed HIV in the country and is struggling with what has been referred to in the news as an ‘unstoppable epidemic’ [40]. The State of California and local stakeholders have responded to this challenge with active prevention programs such as Get Screened Oakland [41,42].

We recruited ten HIV/AIDS CBOs in Los Angeles and Alameda Counties to serve as study sites. All of these agencies are well-established CBOs that serve large numbers of HIV-infected African Americans. In fact, these CBOs expressed particular interest in offering services to couples because they currently do not provide such services. These agencies were identified as having met seven key elements identified in the literature as important in determining agencies’ readiness to implement a new intervention [43]. These include: a respected local community advocate; strong administrative support; formal organizational commitments and stability; commitment of necessary resources to incorporate the program into existing services; program credibility within the community; adequate facilitators/staff; and potential for the program to be self-sustaining or willing to seek additional funding. In addition, all of the agencies have adequate space for conducting private assessment and group sessions.

Approximately 200 staff members from these agencies (~20 per agency) will complete the staff- and organizational-level measures described below. A subset of these individuals (approximately 50 ‘key stakeholders,’ including agency administrators, site coordinators, and facilitators) who are directly involved in implementation will complete semi-structured interviews.

In addition, in order to support potential scale up and spread of the intervention, each of the agencies will be asked later in the project to identify two to three additional agencies that may be interested in learning about Eban II. In the last six months of the study, the Management Team will meet at a retreat with the key stakeholders and representatives from the agencies that they have identified. Preliminary findings will be discussed, and participating agencies will describe their experiences and engage in dialogue about implementation of Eban II.

### Couples-level sample

The study employs multiple community outreach and marketing strategies to recruit and screen 215 to 230 couples, expecting to enroll 180 African American heterosexual couples. The dropout rate in the Eban randomized controlled trial (RCT) was low at 13% in the intervention condition [44]. We conservatively project a 17% to 22% overall attrition rate since this study will be conducted in CBOs rather than in university-based study sites. Eligibility criteria are as follows:

1. Self-identified heterosexuals

2. One partner is HIV-negative, the other is HIV-positive, and partners know each other’s HIV status; serostatus confirmed through written verification from testing facility.

3. At least one partner identifies as African American.

4. Each partner is no younger than 18 and no older than 60.

5. Couple has been in a relationship for at least six months and intends to remain together.

6. Couple reports having unprotected intercourse at least once in the previous 90 days.

7. Couple has no plans to relocate beyond a reasonable distance from the study site.

8. Members of couple are willing to complete the study even if their relationship ends.

### Activities and procedures

Our implementation approach for Eban II is phased to correspond conceptually to the PCM and involves several strategies and tools that support implementation in the participating CBOs (see Table 1).

**Table 1:** Implementation strategies and tools by Program Change Model (PCM) phase

**PCM Phase**	**Tools and strategies**	**Purpose**
**Training**	Eban training manuals	Provides instructions for intervention delivery
	Eban videos	Operationalizes intervention core elements
	Eban Sharepoint	Provides all training tools in accessible format
**Adoption**	HIV Fact Sheets (patient and provider versions)	Educates about HIV prevention
**Adoption**	Project kick-off	Engages sites in project, concretizes expectations
	Site coordinators	Serve as intervention champions and liaisons
**Implementation**	Monthly inter-agency calls	Build sense of communal effort; sustain leadership support
	Continual feedback on implementation	Supports tailoring of implementation strategies
	Technical assistance, especially during sustainability	Promotes collaboration and commitment to sustainability
	Pre-sustainability workshops	Increase likelihood of sustainability
**Practice improvement**	Project wrap-up retreat	Promotes scale up and spread

With these strategies and tools, our goal is to ensure that the intervention and its implementation become sustainable beyond the life of the project, and that participating agencies will be motivated to share their experiences with other agencies that may be interested in serving HIV-serodiscordant couples, *i.e.*, in spreading the intervention across service agencies.

### Phase one: training and pre-implementation (year one)

Three main activities occur in year one: a project kick-off, training, and baseline (developmental) evaluation of staff and organizational factors. As a key strategy supporting implementation, we will hold an official ‘kick-off,’ *i.e.*, a weeklong orientation and training of the program managers, site coordinators, facilitators, agency administrators and staff, and the data collectors. Consistent with the procedures used in the Eban RCT, all facilitators will receive 40 hours of training from certified Eban facilitators. All must demonstrate competency before they are certified as facilitators. In addition, each year, CBO directors and two staff members/agency will have the opportunity to be retrained in order to have fresh skills for the active intervention phase and to maximize their potential for sustainability. Site coordinators and data collectors will receive 10 hours of training and observed practice in their respective region.

### Phases two and three: adoption and implementation (years one to five)

These phases involve: developmental evaluation, delivery of the Eban II intervention, couples-level data collection (aim three), implementation-focused evaluation, progress-focused evaluation, and assessment of costs.

#### Developmental evaluation

Key stakeholders at each CBO will complete pre-implementation interviews regarding motivation, interest, receptivity, expectations, capacity to conduct the project, and existing treatment protocols. Following delivery of each cycle of the intervention, stakeholders will be re-interviewed to inquire about feasibility and barriers. Data from these interviews will be used by the research team to tailor the implementation approach.

### Delivery of the Eban II intervention

Agencies will be activated sequentially as cohorts are completed. The flow of agency participation will be as follows: year one, two agencies; year two, two agencies; year three, three agencies; year four, three agencies. All agencies will be required to complete three Eban cycles (eight weeks/cycle). In year five, three-month follow-up evaluation data collection will continue and sustainability will be studied at the agencies that complete the active phase in year four.

### Screening and enrollment of couples

Initial phone or in-person contact with project staff will be made by the HIV-positive partners who will be screened to determine their eligibility. If eligible, they will inform their partner who will contact the staff and be screened to confirm their eligibility. Consented couples will be scheduled for the baseline assessment. Sociodemographic information on couples who do not meet criteria or refuse to participate and reasons for non-eligibility or refusal will be collected to permit comparisons between eligible participants and non-participants. The assessment battery will be repeated at the posttest and three-month follow-up. If couples break up or a partner is incarcerated or dies, the remaining individual will be followed throughout the three-month follow-up. These individuals will receive a ‘break up’ curriculum individually and will be assessed in order to examine their adherence to safer sex behaviors with new partners. Couples will be randomly assigned in a 2:1 ratio to either Eban II active treatment or to waitlist control (WL), with 2/3 (n = 118) of the couples assigned to Eban II and 1/3 (n = 62) assigned to WL in a cross-over design for group comparisons. At the end of the three-month follow-up, couples assigned to WL will be offered the intervention and their three-month data will serve as their baseline for the treatment analyses. During their time on the waitlist, couples will be called weekly by the site coordinators to ensure that they are receiving standard care.

### The Eban II intervention

This eight-week, standardized, manualized intervention is facilitated by a male–female team. The intervention (see Table 2) involves: strategies that address the broad array of factors influencing risk behavior among HIV-affected couples; dyadic and group processes that take advantage of relationship and group dynamics; and educational and culturally appropriate sessions [45].

**Table 2:** Core elements of the Eban II intervention

**Sessions**	**Core elements**
**Sessions**	**Principles**
1. ‘Preparing for the Journey’ (Group Session)	Gender, Ethnic, and Cultural Pride
2. ‘Enhancing Couple Communication’ (Couple session)	The Talk and Listen Technique
	Triggers to Risky Behavior
	Learn to Problem Solve with ‘FENCE’
	Identifying Sexual Abuse
3. ‘Tools for the Journey’ (Couple session)	Condom Use
4. ‘Sharing the Load’ (Couple session)	Gender, Ethnic, and Cultural Pride as Couples
5. ‘It Takes a Village’ (Group session)	Reframing Difficult Situations
6. ‘Strengthening the Village’ (Group session)	Self Talk and Relapse Prevention
7. ‘Expanding the Village’ (Group session)	Networking with others and sexual communication with partners
8. ‘Celebrating our Relationship’ (Couple session)	A review of Nguzo Saba principles of unity and rededication to protecting each other

Unlike a traditional RCT, individuals in this study will not be incentivized for attending sessions. However, our experience indicates that incentives enhance attendance and active participation; community agencies frequently provide such incentives to enhance participation in routine programs. Therefore, we will provide minimal participation incentives similar to those often provided routinely by the agencies, such as bus tokens and gift cards at pre-, post-, and three-month follow-up. In addition, usual care, services, and referrals will be available to the WL couples while they wait to enter the risk reduction program. We will learn more about the impact of incentives during sustainability, when these incentives are no longer provided.

### Couples-level data collection procedures

The self-report assessment developed for Eban and pre-programmed into Audio Computer-Assisted Self-Interview (ACASI) [46] takes about 90 minutes to complete. ACASI provides both audio and video presentation of the questions and response options on a laptop computer. ACASI has been shown to significantly decrease social desirability bias [21].

### Implementation-focused evaluation

Several types of assessments will be used to track implementation processes, specifically with regard to fidelity, dose, and intensity of intervention. Throughout the intervention, we will monitor: session attendance, session completion, and participant satisfaction with sessions. Facilitators will also complete short surveys noting adherence to the curriculum, level of participation, specific obstacles that may have arisen, and what components appeared to be most/least appropriate for the session’s participants. Facilitators will also complete overall ratings of each couple’s engagement, competency, and knowledge. To assess fidelity, sessions will be digitally recorded. For the first intervention cohort at each site, all recordings will be reviewed and feedback will be provided to facilitators. Subsequently, a random 15% sample of all session recordings will be reviewed and scored for fidelity to core elements, with a criterion of 80% or more of the total elements considered acceptable [47].

### Progress-focused evaluation

At the organizational level, this component will involve assessing the monthly SCIN conference calls. Minutes of all calls will be maintained (including attendance rosters), as will minutes from all project-related meetings. Regarding progress at the couples-level, this component will involve weekly calls between the regional project managers and the site coordinators. A periodic onsite review of procedures at each site will be conducted, and a random selection of 5% of pre- and post-test and follow-up interviews for each site coordinator and data collector will be reviewed to ensure that the protocol is administered in a standardized fashion.

### Cost assessment

The cost of the delivered services will be calculated using a customized cost-analysis spreadsheet which uses state of the art recommendations for cost analysis as articulated by the US Panel on Cost-Effectiveness in Health and Medicine [48]. The cost analysis data will be collected by the data collectors and sent on a quarterly basis to the management team, and reviewed with the cost consultant in quarterly conference calls to analyze the cost information, identify trends, and solve any problems with data collection.

### Phase four: practice improvement

Phase four involves interpretive evaluation, assessment of sustainability, and a project retreat. Because this project will occur in waves, practice improvement will be examined after each agency completes the active intervention phase (delivery of the groups and completion of three-month follow-up). Sustainability will be examined for nine months following the completion of the required three groups and follow-ups.

### Interpretive evaluation

Key stakeholders will have completed interviews regarding their satisfaction with Eban II during active implementation, and their expectations regarding sustainability and cost effectiveness. Following sustainability, these stakeholders will be re-interviewed.

### Sustainability

We define the sustainability phase as beginning after the active implementation phase is completed at each site (*i.e.*, baseline through the 3-month follow-up). At this point, the reliance on grant funds ends and sites will be encouraged to integrate Eban II into their usual services. At each site, the nine-month sustainability period will be preceded by a pre-sustainability workshop to identify and address any issues that the agencies perceive to be barriers [49]. During sustainability, Eban II will be delivered in the CBOs with trained staff serving as co-facilitators, supported by technical assistance (including quality assurance) from the management team. Technical assistance will include retraining in the intervention, sharing resources, offering suggestions on lessons learned, and review of session tapes to assess fidelity. Agencies that discontinue participation during this phase will be contacted to encourage participation and/or to identify the reasons for discontinuation. Agencies that independently deliver two eight-week cycles of the intervention with two to three couples in each cycle and maintain fidelity to the intervention core elements will be considered to have achieved sustainability. Pre-post couples-level measures will also be collected in order to examine outcomes. Sustainability will be assessed by documenting the number of couples that each agency recruited and enrolled in Eban II; the number of couples who completed the intervention; and how much technical assistance they needed and received.

### Project retreat

Additional funds will be sought to support a project retreat for the management and implementation teams to come together with additional representatives from the SCIN to discuss preliminary findings and the future of the intervention. Individuals from interested agencies that were not part of the original 10 CBOs will be invited to attend a designated portion of this retreat, and a structured discussion guide will be used to explore likelihood of adoption, perceived need for the intervention, preliminary perceptions of the program, and its trialability.

### Measures to be completed by staff

An electronic Staff Survey will capture basic demographics of staff (~20 per agency) including education level and professional experience. The 50-item Evidence-Based Practices Attitudes Scale (EBPAS) [50] will be used to assess staff attitudes toward evidence-based practices. The widely used Maslach Burnout Inventory (MBI; Maslach) [51] will be used to assess staff experience of workload. Subscales from the TCU Survey of Organizational Functioning (TCU SOF) [52] will be used to assess motivational factors, program resources, staff attributes, and organizational readiness. All subscales of the SOF have demonstrated good internal consistency and validity [53].

In addition to these structured measures, semi-structured interviews will be conducted with a subset of key stakeholders (approximate n = 50; five per agency). Process-focused interviews will include questions about feasibility, satisfaction, perceived fidelity, barriers, desired revisions, perceived cost-effectiveness, and expectations for sustainability. Sustainability-focused interviews will include similar questions, but will refer to the sustainability period and their satisfaction with and future expectations of the utility and cost-effectiveness of Eban II as a long-term option for agencies’ usual menu of services.

### Cost analysis measures

We will use a cost-analysis spreadsheet approach to measure the cost of HIV prevention, case management, and adherence services for CBOs providing front-line services. The spreadsheet incorporates key cost analysis principles, calculates the cost per client of HIV/AIDS services delivered, and also conducts threshold analyses to set performance standards for how many HIV infections would have to be averted for Eban II to be considered cost-saving or cost-effective to society.

### Measures to be completed by clients

Primary behavioral and physiologic outcomes are as follows:

1. Percentage of condom-protected sexual acts in the past 90 days.

2. Number of episodes of unprotected sexual intercourse.

3. Number of sexual partners.

4. Types of partners (primary, casual, new).

5. Frequency of oral, anal, and vaginal sex.

6. Use of alcohol and other drugs prior to sexual intercourse.

7. Partners’ risk status (*e.g.*, sexual partner has other partners).

We will also track concurrency, seroconversion, and incident STIs. In order to control for response bias, several steps will be taken, including: assessing sexual behaviors over a relatively brief period (*i.e.*, past three months at baseline and follow-ups and past seven weeks at post-intervention); providing participants with a calendar for benchmarking salient dates; involving only trained data collectors in the data collection; using the ACASI; and administering a five-item measure of social desirability bias [54].

Potential covariates include demographic characteristics; mental health status as measured by the 45-item Brief Symptom Inventory (BSI); history of sexual abuse as measured by the Wyatt Sex History Questionnaire (WSHQ) [55]; and history of physical abuse as measured by the 19-item Revised Conflict Tactics Scale (CTS) [56], which assesses both history and recent (*i.e.* past 90 days) experiences of abuse. Hypothesized mediators [57] include condom use self-efficacy, which has been linked to effective condom use [58,59], as measured by the Condom Use Self-Efficacy Scale [60]; condom communication self-efficacy as measured by a nine-item scale measuring confidence in negotiating the use of a male condom [60]; and couples’ sexual communication skills as measured by a seven-item scale on the level of reported comfort on safer sex communication items [61]. Moderators include several variables identified in Eban I as likely to moderate intervention effects [57]: the length of the couple’s relationship, because those in longer relationships are less likely to practice safer sex [45]; relationship satisfaction will be assessed with the Dyadic Adjustment Scale [62], which asks how partners relate to each other about family finances, time spent together, relational factors, and intimacy; substance use/abuse (frequency and amount of alcohol use each day in the past three months and age of first use) as measured by the CAGE [63] to assess dependence on alcohol, and section B of the NIDA Risk Behavior Assessment (RBA) [64] to assess the frequency, modality, and level of use of licit and illicit drugs in the past month.

### Data management and analysis procedures

#### Quality control procedures

Quality control procedures include quality controls during the training of the co-facilitator teams; and during the running of groups, including checks of a random sample of audiotaped sessions and providing supervision and corrective feedback as needed. Co-facilitator teams will be asked to complete process notes at the end of each session, and couples will complete satisfaction ratings at the end of each session. Co-facilitator teams, site coordinators, and couples will also be asked to provide an overall evaluation of perceived effectiveness and their level of overall satisfaction with the intervention at the posttest assessment session.

### Sample size calculation and power analyses

Sample size calculation and power analyses were conducted at both the agency- and couple-levels to ensure that we have sufficient statistical power for all analyses. At the agency level, the power analysis was based on the comparisons of key measures from providers (*e.g.*, provider attitudes toward evidence-based practices). From the 10 selected agencies, we will collect data from 100 non-clerical staff (10 providers per agency) in order to provide sufficient power to examine the within- and between-agency variation. With a type I of 0.05, type II error of 0.2 (or power of 80%), intra-agency correlation at 0.15 level, 100 raters from the 10 agencies will enable us to detect an effect size as small as 0.9 in standard deviation unit for measures from staff [65,66]. Non-parametric and parsimonious models will be used to accommodate the limitation of a relatively small number of agencies.

At the couples level, calculations were carried out based on the comparison of key outcome measures of incidents of unprotected sex and an increase in the proportion of condom use (*i.e.*, the number of times condoms were used during intercourse divided by the number of times sexual intercourse is reported) between intervention and waitlist control. With a repeated measures design, type I error 0.05, type II error 0.20, intra-couple correlation 0.4, the mean number of available repeated measures 2.5 (from baseline, post and three-month measurements), and a 2:1 ratio of intervention and waitlist control, a total of 174 couples would allow us to detect an effect size as small as 0.30 in standard deviation unit for number of unprotected sexual intercourse acts [65,67]. Thus, our planned sample of 180 couples provides sufficient power.

### Data analysis plan

#### Specific aim one

Qualitative data will document how we assist the 10 CBOs to implement and sustain the intervention. As a broad implementation aim, there are no hypotheses associated with this aim. Instead, the focus will be on describing the process of assisting the CBOs with implementation and sustainability. This description and summary will be generated from interviews and notes from the monthly SCIN calls, along with other qualitative data that is gathered throughout the study.

#### Specific aim two

To document the implementation process and identify barriers and facilitators to adoption, fidelity, and sustainability, we will utilize a variety of analytic approaches. First, marginal distributions of each of the measures at the agency level (*e.g.*, intervention cohorts completed, provider attitudes, etc.) will be obtained. For continuous measures (*e.g.*, the number of facilitator teams trained), we will calculate the range, mean, median, quartiles, and standard deviations. For categorical measures (*e.g.*, fidelity to core elements), frequency distributions and mode will be obtained. An ordinal measure that reflects the number of intervention groups conducted at each agency will be created, which will be used to link with the number of intervention cycles completed and to test the dose–response of the intervention. For performance measures (*e.g.*, satisfaction) obtained from providers at the CBOs, analyses will be conducted, and within and between agency variations will be examined. Non-parametric methods (*e.g.*, Mann Whitney test) will be used to accommodate the relative small sample size of agencies. Rigorous statistical tests will be conducted in three steps: marginal distributions of the number of intervention cycles completed will be obtained; two-way table chi-squares or analysis of variance will be used to evaluate the relationships between the categories of number of intervention cycles completed and the extent of successful implementation, which is measured by an array of variables (*e.g.*, number of couples served, degree of fidelity, level of satisfaction with the intervention, etc.); and multivariate analyses will be conducted through regression models to examine the relationships between provider measures and number of intervention cycles completed, controlling for other agency characteristics such as agency size, number of staff, size of budget, etc.

Data regarding acceptability, barriers, and facilitators will be derived from interviews and implementation-focused evaluation measures. As we are examining implementation in 10 large, diverse CBOs, barriers and facilitators will likely vary by agency. The qualitative data will provide the research team with extensive information about acceptability, barriers, and facilitators. In addition, we will examine the facilitator and couples’ satisfaction ratings to characterize acceptability of the intervention. These ratings will be compared both within and across agencies. Potential determinants of adoption and fidelity include but are not limited to training factors, competency at delivering the intervention, types of facilitators (*e.g.*, whether agencies used their own staff as facilitators or used the floating facilitator team), couple ‘mix’ in a given cohort, retention, and satisfaction. All of these factors will be examined when characterizing fidelity across agencies. In addition, based on interviews with facilitators and clients, we will remain open to other determinants that may emerge during implementation. There are several possible determinants of sustainability. We hypothesize that sustainability will be achieved in agencies that have their own trained staff facilitators who deliver the intervention with fidelity and who had positive experiences with the intervention during the implementation phase. To test this hypothesis, we will perform analyses in two levels: (1) Bivariate analyses between sustainability and characteristics of the CBOs and staff. The Pearson Chi-square will be used to test association. ANOVA will be used to test the means of the continuous measures (*e.g.*, ‘the number of agencies that continue to implement Eban II with fidelity’) across the different levels of a categorical measure (‘Does your agency have specific services for high-risk or HIV-positive couples? Yes/no’) at each time point of data collection; (2) Longitudinal analyses for long-term effects. Generalized linear mixed models (GLMM) [67,68] will be used to evaluate the relationship between sustainability and characteristics of the CBOs. GLMM cannot only model global fixed effects (*e.g.*, the number of full-time staff), but also can model random variation (*e.g.*, change over time of individual agency), which is particularly useful.

### Qualitative data analysis—aims one and two

All interviews will be digitally recorded and transcribed by trained staff. ATLAS.ti will be used for qualitative analysis. Using the constant comparison analytic approach [69], a preliminary codebook will be developed both inductively and deductively from a sub-sample of interviews within and across agencies at baseline. Qualitative findings at baseline will be augmented by preliminary analyses of staff-level data from the structured measures described above (*e.g.*, burnout, attitudes toward evidence-based practices, etc.), and a baseline profile will be developed for each agency. These profiles will be used to tailor implementation at each site. This approach of using baseline data as diagnostic and informative for tailored implementation has been employed by the lead author (AH) in a prior implementation study [29]. The codebook will be elaborated upon and adjusted as each round of interviews is reviewed until thematic saturation is achieved within and across cycles of interviews. Interviews will be compared within each agency, across agencies, across different types of respondents, and over time. Additional sources of qualitative data (*i.e.* meeting minutes, archival information) will also be included in the data set. We will analyze the data specifically for barriers to and facilitators of implementation, including but not limited to the ways in which the project’s strategies and tools affect adoption, fidelity, and sustainability. In addition to identifying themes and patterns qualitatively, we will examine statistical associations between important process and outcome variables such as satisfaction with the intervention, fidelity, and retention, and improvement in behavioral outcomes. Agency profiles will be revisited and further developed at the end of the active implementation phase, and again after sustainability.

### Specific aim three

We will use repeated measures regression models and multi-level hierarchical modeling [70,71] to evaluate the effect of characteristics of agencies, staff, and couples on the behavioral and psychosocial outcomes. For repeated measures regression models, we will directly incorporate measures into models and deal with intra-class correlations from agency and staff through covariance matrix. With the multi-level nested structure from individuals, couples, agencies, and staff, the hierarchical models will be fitted in three possible steps: (a) to set the foundation for a class of hierarchical models, first we will construct individual-couple-level models with repeated measurements (baseline, post and three-month) within a couple that involves time (and intercept); (b) we will then construct across the couples-level models that involve explanatory variables such as couple demographics; health history; history of alcohol and recreational drug use; HIV/AIDS risk reduction knowledge; perceived couple sexual norms; intervention components, and a binary variable indicating immediate active treatment or wait list control; (c) we will model the regression coefficients obtained from step b at the agency level as functions of explanatory variables of agency characteristics (*e.g.*, organization size and budget, number of staff, etc.). Finally, we will then combine models from steps a, b, and c to form the complete model, which will be estimated through special procedures and software, such as WinBUGS and MlwiN [72,73].

### Secondary aim

To determine the cost-effectiveness of implementation of Eban II, standard cost-effective analysis will be conducted using health economic analysis approaches. Two-staged models (instrumental variable regression) [74,75] will be applied and implementation costs and potential costs saving through the intervention will be estimated.

## Trial status

Data collection is underway. Data cleaning and analysis have not commenced.

## Discussion

This hybrid implementation/effectiveness study has the potential to illuminate the processes and complexities associated with supporting adoption of an evidence-based program in resource-limited organizations that typically serve underserved, economically disadvantaged individuals. The study’s innovative features include the dynamic wait-listed design [37], the sustainability period (not typically included in implementation trials) [35], the real-world effectiveness conditions under which the intervention is being tested, and the participatory approach to implementation [76]. One of the overarching goals of the study is to ‘dive deep’ into the cultures not only of the organizations but also of their surrounding communities, which have been greatly impacted by HIV and by health disparities in general, especially due to shrinking state and national budgets designated for HIV prevention efforts in these communities. In systematically addressing current community-, organizational-, and client-level complexities with an innovative and partnered design, this study exemplifies the five core values of implementation science: rigor and relevance, efficiency, collaboration, improved capacity, and cumulative knowledge [44].

## Competing interests

The authors declare that they have no competing interests.

## Authors’ contributions

AH serves as a Co-Investigator and was involved in conception and design of the manuscript, and drafted the manuscript. BM serves as a Co-Investigator and was involved in conception and design of the manuscript, and critically reviewed the manuscript for important intellectual content. JW serves as a Co-Investigator and was involved in conception and design of the manuscript, and critically reviewed the manuscript for important intellectual content. HL serves as a Co-Investigator and was involved in conception and design of the manuscript, and critically reviewed the manuscript for important intellectual content. AE was involved in conception and design of the manuscript, and critically reviewed the manuscript for important intellectual content. CH was involved in conception and design of the manuscript, and critically reviewed the manuscript for important intellectual content. GW serves as Principal Investigator of the study and was involved in conception and design of the manuscript, and critically reviewed the manuscript for important intellectual content. All authors read and approved the final manuscript.
